# Climatic regions as an indicator of forest coarse and fine woody debris carbon stocks in the United States

**DOI:** 10.1186/1750-0680-3-5

**Published:** 2008-06-09

**Authors:** Christopher W Woodall, Greg C Liknes

**Affiliations:** 1 USDA Forest Service, Northern Research Station, 1992 Folwell Avenue, St. Paul, MN 55108, USA

## Abstract

**Background:**

Coarse and fine woody debris are substantial forest ecosystem carbon stocks; however, there is a lack of understanding how these detrital carbon stocks vary across forested landscapes. Because forest woody detritus production and decay rates may partially depend on climatic conditions, the accumulation of coarse and fine woody debris carbon stocks in forests may be correlated with climate. This study used a nationwide inventory of coarse and fine woody debris in the United States to examine how these carbon stocks vary by climatic regions and variables.

**Results:**

Mean coarse and fine woody debris forest carbon stocks vary by Köppen's climatic regions across the United States. The highest carbon stocks were found in regions with cool summers while the lowest carbon stocks were found in arid desert/steppes or temperate humid regions. Coarse and fine woody debris carbon stocks were found to be positively correlated with available moisture and negatively correlated with maximum temperature.

**Conclusion:**

It was concluded with only medium confidence that coarse and fine woody debris carbon stocks may be at risk of becoming net emitter of carbon under a global climate warming scenario as increases in coarse or fine woody debris production (sinks) may be more than offset by increases in forest woody detritus decay rates (emission). Given the preliminary results of this study and the rather tenuous status of coarse and fine woody debris carbon stocks as either a source or sink of CO_2_, further research is suggested in the areas of forest detritus decay and production.

## Background

Estimation of carbon sequestration using large-scale forest inventory data has become important due to the link between possible climate change and the accumulation of greenhouse gases in the atmosphere [1,2]. In 1992, 150 countries including the U.S. signed the United Nations Framework Convention on Climate Change that resulted in the development of annual reports of greenhouse gas inventories including carbon in forests. Forest carbon pools are often delineated as standing live trees, standing dead trees, down and dead woody materials, forest floor, understory, and soils. The down and dead woody materials pool (detritus) essentially consists of coarse woody debris, fine woody debris, and stumps. Coarse woody debris is defined by the Forest Inventory and Analysis (FIA) program of the USDA Forest Service as down and dead woody material at least 7.62 cm in diameter [3]. Fine woody debris is defined by FIA as dead and down woody material with a diameter between 0.01 and 7.61 cm [3]. In the U.S., it has been estimated that 35 % of the total forest carbon pool is in live vegetation, 52 % is in the soil, and 14 % is in dead organic material [4]. Therefore, estimating coarse and fine woody debris carbon stocks across the United States is crucial to national carbon reporting and monitoring.

Forest terrestrial carbon sinks represent a fine balance between the influx of CO_2 _into photosynthesis and the efflux of CO_2 _through woody decay processes [1]. The decay rate of any individual piece of forest dead wood is determined by substrate quality, microbial activity, air temperature, and available moisture [5]. Similarly, the productive capacity of any given forest is partially governed by climatic variables such as temperature [6]. Some studies have suggested that forest detritus production and decay may be in balance [7], whereas others have suggested increased detritus decomposition rates may ultimately cause forest detritus carbon pools to become net CO_2 _emitters [8,9]. Quantifying the dynamics of forest detritus carbon accumulation and turnover within a scenario of global climate warming is critical to predicting the future inventory of United States carbon stocks. Indeed, some studies have already indicated the effects that changing climate can have on the terrestrial carbon cycle [10] and highlighted the possibility of increasing emission of CO2 from non-live forest carbon pools such as soils [11]. Emerging suggestions to bury coarse woody debris as a cost effective carbon sequestration technique [12] would be impacted if coarse woody decay rates are increased by changing climate. To date, initial investigations of coarse and fine woody debris carbon stocks across classes of latitude have indicated that these carbon stocks may be related to climatic variables [13]. Therefore, correlating coarse and fine woody debris carbon stocks with climatic regions and variables across the United States is highly warranted.

The goal of this study is to relate forest coarse and fine woody debris carbon stocks to climatic regions and variables across the United States with specific objectives including: 1) to estimate mean coarse woody debris, fine woody debris, and total woody detritus carbon stocks (coarse + fine woody debris) by Köppen climatic regions, 2) to correlate/model coarse woody debris, fine woody debris, and total woody detritus carbon stocks with individual climatic variables (average annual precipitation (PRECIP), mean annual maximum temperature (TMAX), mean annual minimum temperature (TMIN), moisture index (MOIST), variability cause (VAR), and potential evapotranspiration (EVAP)), and 3) to interpret the results of this study in the context of possible climate change.

## Results

**Figure 1:**
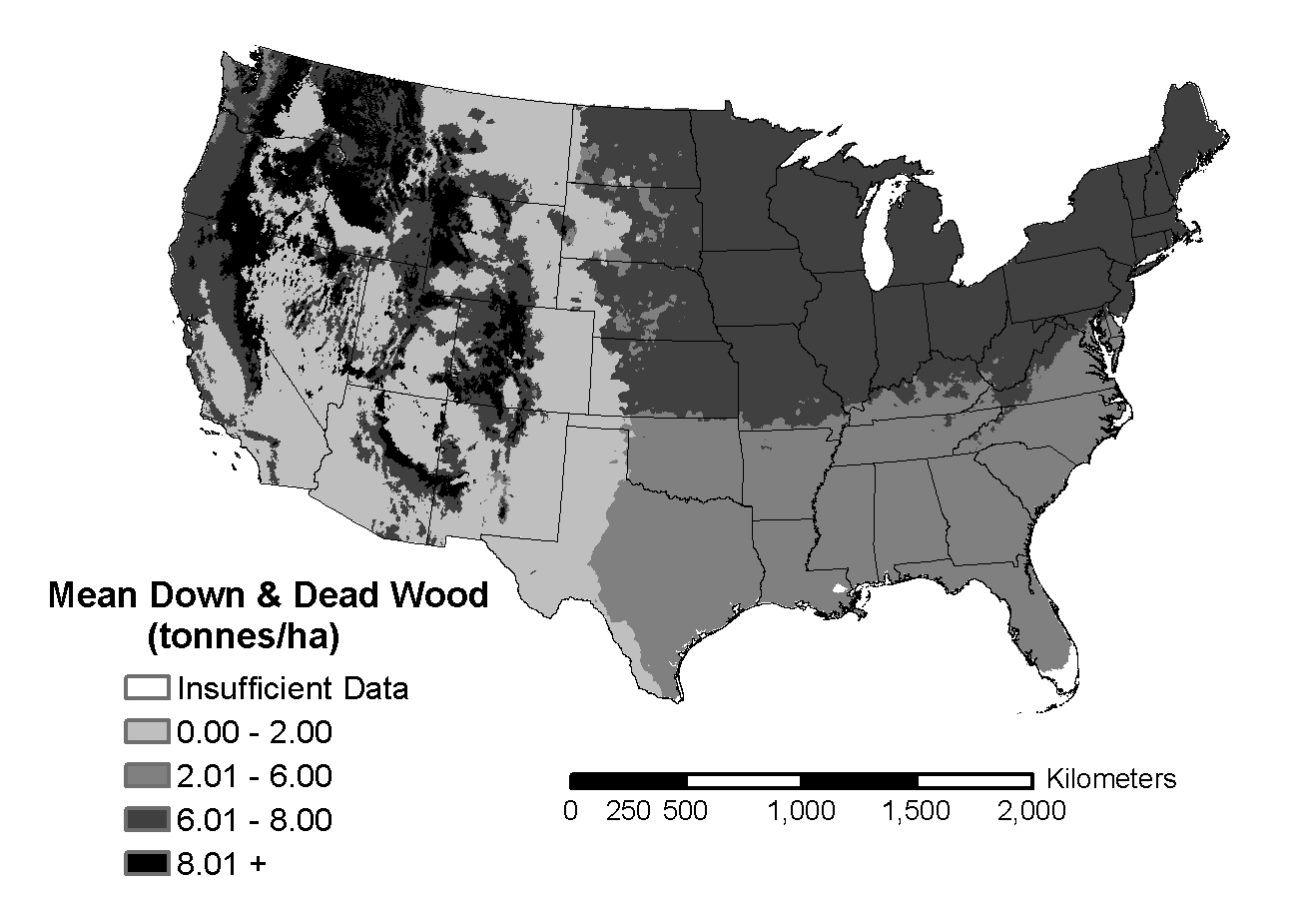
Continental U.S. Köppen climatic regions shaded by classes of mean total down woody C stocks.

Coarse and fine woody debris carbon stocks vary by climatic region with the largest mean stocks found in dry/cool climates such as "warm temperate with dry summer (Cs)" and "snow, fully humid, cool summer (Dfc)" where decay rates may be reduced (Table 1). Forest ecosystems associated with these climatic regions are chaparral, northern Rockies, and extreme northern boreal. Climatic regions with the lowest mean coarse and fine woody debris carbon stocks were dry/hot climates such as "arid desert (BW)" or "warm temperate, fully humid (Cf)." Forest ecosystems associated with these climatic zones are southwestern deserts and steppes or southeastern mixed forests. When displayed spatially, climatic regions that are cooler (i.e. higher latitudes or higher elevation) tend to have higher downed and dead wood forest carbon stocks (Fig. 1). ANOVA's indicated significant differences between the carbon stock means among climatic regions (p-value < 0.0001).

**Table 1:** Means and associated standard errors for forest coarse woody debris (CWD), fine woody debris (FWD), and total down woody carbon (C) stocks (tonnes ha^-1) for Köppen's climatic regions/subgroups across the United States

Climatic region	n	Mean CWD carbon (tonnes ha^-1)	Std err.	Mean FWD carbon (tonnes ha^-1)	Std err.	Mean total down woody C (tonnes ha^-1)	Std err.
BS	591	0.47	0.07	0.89	0.10	1.36	0.13
BW	109	0.00	0.00	0.08	0.06	0.09	0.06
Cs	583	5.21	0.36	2.57	0.14	7.78	0.43
Cf	1835	2.24	0.11	2.94	0.07	5.18	0.14
Ds	609	5.70	0.34	2.59	0.12	8.29	0.41
Dw	9	4.05	1.36	1.29	0.64	5.33	1.71
Dfa	676	3.43	0.21	3.16	0.12	6.59	0.27
Dfb	1494	4.27	0.15	3.43	0.09	7.69	0.18
Dfc	188	7.11	0.55	2.62	0.17	9.73	0.59
H	9	3.78	1.84	3.85	1.44	7.63	2.41

When correlated with a host of individual climatic variables, coarse and fine woody debris carbon stocks were found to be positively correlated with PRECIP and MOIST. In contrast, coarse and fine woody debris were negatively correlated with TMAX, TMIN, VAR, and EVAP (Table 2). In terms of total down woody carbon, the strongest correlations were with MOIST (cor. coeff. = 0.36) and TMAX (cor. coeff. = -0.30). Fine woody debris carbon stocks had weaker correlations with climatic variables when compared to coarse woody debris carbon stock and climatic correlations.

**Table 2:** Pearson's correlations coefficients and associated p-values for forest coarse woody debris (CWD), fine woody debris (FWD), and total down woody carbon (C) stocks by climatic attributes

Climate variables	Coarse woody debris C		Fine woody debris C		Total down woody C	
	
	Corr. coeff.	p-value	Corr. coeff.	p-value	Corr. coeff.	p-value
PRECIP	0.25	< 0.001	0.22	< 0.001	0.29	< 0.001
TMAX	-0.30	< 0.001	-0.13	< 0.001	-0.30	< 0.001
TMIN	-0.24	< 0.001	-0.07	< 0.001	-0.22	< 0.001
MOIST	0.29	< 0.001	0.29	< 0.001	0.36	< 0.001
VAR	-0.19	< 0.001	-0.10	< 0.001	-0.19	< 0.001
EVAP	-0.25	< 0.001	-0.06	< 0.001	-0.23	< 0.001

: 30-year mean annual yearly precipitation (PRECIP), 30-year mean daily maximum temperature (TMAX), 30-year mean daily minimum temperature (TMIN), moisture index (MOIST), variability cause (VAR), and potential evapotranspiration (EVAP)

**Figure 2:**
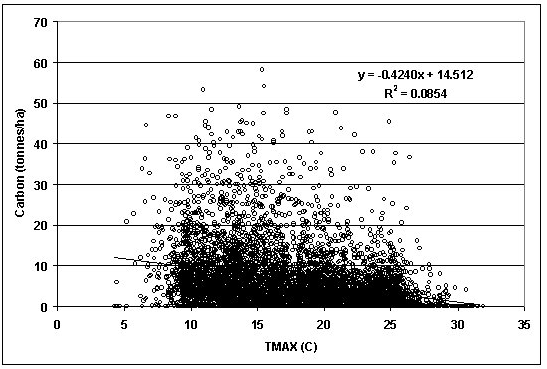
Total down woody C stocks by 30-year mean daily maximum temperature (TMAX) (PRISM Group, 2004).

**Figure 3:**
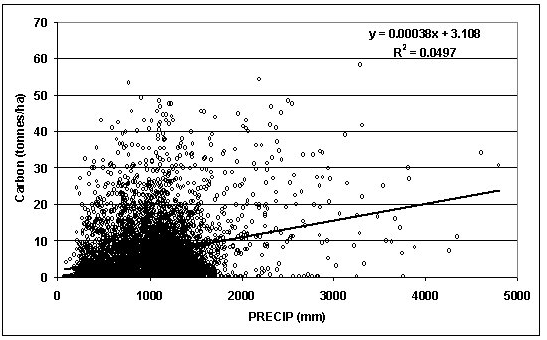
Total down woody C stocks by 30-year mean annual precipitation (PRECIP) (PRISM Group, 2004).

Linear regressions between total woody detritus stocks and TMAX and PRECIP indicated tremendous data scatter along with very weak R-square's (< 0.1) (RMSE TMAX model = 7.386; RMSE PRECIP model = 7.529) (Figs. 2, 3). Total woody detritus stocks had a negative relationship with increasing TMAX. In comparison, total woody detritus stocks had a barely positive relationship with increasing PRECIP. Given that the TMAX and PRECIP variables had some of the stronger correlations with woody detritus carbon stocks, regressions conducted between all remaining dependant dead wood carbon stock variables and independent climatic variables demonstrated very weak relationships and did not contribute beyond correlation results.

## Discussion

This study found that coarse and fine woody debris carbon stocks varied by climatic regions and variables. However, these results were relatively weak and may be partially confounded by the varying levels of decay resistance across the range of tree species in the United States [14,15]. Therefore, the results of this study provide very limited inference potential at small scales (e.g. states), but may help elucidate the dynamics between coarse/fine woody debris carbon stocks and climate at the continental scale. Forest woody detritus carbon stocks are found in the largest amounts in climates that are moist enough to support productive forests while at the same time are cool enough to reduce decay rates. This hypothesis was evidenced by the "snow, fully humid, cool summer" climatic region (Dfc) having the highest mean total dead and downed wood carbon stock of 9.73 tonnes ha^-1. The climatic region with the lowest mean forest dead and downed wood carbon stock was that of "arid desert" (BW) where the hot temperatures increase decay rates and the lack of moisture reduces forest productivity. These results are similar to those found at smaller scales [9,16-18].

Coarse and fine woody debris carbon stocks vary individually with climatic regions/variables. The cool, moist climatic regions of the Pacific Northwest and high elevations in the Rocky Mountains have the greatest disparity between coarse and fine woody debris carbon stocks with coarse woody debris stocks exceeding fine woody debris stocks by 4.45 tonnes ha^-1 on average. In contrast, the humid and hot climatic regions in the southeastern United States had fine woody debris carbon stocks exceeding coarse woody debris stocks by 0.70 tonnes ha^-1 on average. Furthermore, fine woody debris carbon stocks were less correlated with climatic variables than coarse woody debris carbon stocks. Given the ephemeral nature of fine woody debris carbon stocks with their rapid decay and turnover, it is proposed that fine woody debris carbon stocks may be relatively unaffected by increases in global temperature. Studies at smaller scales proposed that forest litter carbon stocks were unaffected by changes in temperature [1,7,19,20]. Future fine woody debris carbon stocks may be more affected by tree species shifts than climate shifts [20], an indirect effect of global climate change.

The relationship between temperature, moisture, and forest woody detritus carbon stocks is complicated and not easily summarized [15]. Increases in temperatures and/or moisture are required for increases in forest productivity and subsequent forest detritus accumulation. However, increased temperatures and moisture increase decay rates. Therefore, the climatic variables that increase forest woody detritus production also increase forest detritus decay. Additionally, forest woody detritus resistance to decay is tree species-specific [15], and tree species spatial distributions are partially dependent on climate in the United States [21]. Finally, differences in stand age, disturbance events, and silvicultural practices may confound analyses. Some studies have found forest attributes such as management practices and disturbance history to have an effect on coarse and fine woody debris biomass at small scales [22,23] while others have found little correlation between these variables and woody detritus at large-scales [24,25] such as examined in this study. Caveats aside, this study found that forest woody detritus carbon stocks were almost equally correlated with maximum temperature and moisture in opposing directions. This study supports the hypothesis proposed by other studies [1,7-9,16,26] that there is a fine balance for some carbon pools (e.g. forest detritus and soils) as a net CO_2 _source or sink, all partially dependent on climate and feedback mechanisms.

Based on this study's results, a few conjectures may be postulated. Under a global warming scenario, forest woody detritus carbon stocks might experience accelerated decay/turnover becoming a net CO_2 _source unless there are concomitant increases in climatic variables favorable to forest productivity (e.g. increased moisture) or reduced detritus decay (e.g. increased CO_2_; see [5]). Forest woody detritus carbon stocks may be similar in nature to northern peatland soil carbon stocks, at risk to accelerated rates of decay and net CO_2 _emission as global temperatures increase [27]. Overall, given the uncertainty in predicting future precipitation and management/mortality events, it can only be conjectured that forest woody detritus carbon stocks may face increased risk of becoming a CO_2 _emitter if temperatures increase [1,8].

## Conclusion

The results of this study established an initial link between forest coarse/fine woody debris carbon stocks and climatic regions/variables. Although this study found the relationships between these carbon stocks and climate to be only moderately evident, we propose that carbon sequestration by forest detritus may be increased by increased moisture availability and reduced by warmer temperatures. A delicate balance may exist where slight changes in climate could change a region's forest woody detritus status as a either a net CO_2 _source or sink. We further conjecture that under a scenario of global warming, forest woody detritus carbon stocks (especially coarse woody debris) will be at risk for becoming net CO_2 _emitters. Given the uncertainty found with all relationships identified in this study, further analysis of this study's data [28] may delineate the influence of the factors (e.g., forest management practices) that probably obscure widely recognized effects of temperature and precipitation on regional/local woody detritus carbon stocks.

## Methods

### CWD and FWD inventory field methods in the U.S

The FIA program, the only congressionally mandated national inventory of U.S. forests, conducts a 3-phase inventory of forest attributes of the country [29]. The FIA sampling design is based on a tessellation of the United States into hexagons approximately 2,428 ha in size with at least one permanent plot established in each hexagon. In phase 1, the population of interest (forest cover) is stratified and plots are assigned to strata to increase the precision of estimates. In phase 2, tree and site attributes are measured for forested plots established in the 2,428-ha hexagons. Phase 2 plots consist of four 7.32-m fixed-radius subplots on which standing trees are inventoried.

In phase 3, a 1/16 subset of phase 2 plots are measured for coarse and fine woody debris on transects radiating from each FIA subplot center [3]. As defined by FIA, coarse woody debris are down logs with a diameter ≥ 7.62 cm along a length ≥ 0.91 m. Information collected for every coarse woody debris piece intersected on each of three 7.32-m transects on each FIA subplot are transect diameter, length, small-end diameter, large-end diameter, decay class, and species. Transect diameter is the diameter of a down woody piece at the point of intersection with a sampling transect. Length is the length of each coarse woody debris piece between the small- and large-end diameters. Decay class is a subjective determination of the amount of decay present in an individual log. Decay class one is the least decayed (freshly fallen log), while decay class five is an extremely decayed log typically consisting of a pile of brown, cubicle rot. The species of each fallen log is identified by determining species-specific bark, branching, bud, and wood composition attributes (excluding decay class five coarse woody debris pieces) (for sample protocol details, see [3,30,31]).

Fine woody debris are sampled on one transect on each subplot. Fine woody debris pieces with diameters less than 2.54 cm were tallied separately on a 1.83-m slope distance transect. Fine woody debris pieces with transect diameters of 2.55 to 7.59 cm were tallied on a 3.05-m slope-distance transect (for more information on class definitions see [32]). Fine woody debris sampling methods on FIA plots are detailed by Woodall & Monleon [3].

### Data

Between 2001 and 2005, a total of 4,531 forest inventory plots were measured for coarse and fine woody debris by the FIA program in 45 of the continental 48 states (sampling not established in Mississippi, Wyoming, and New Mexico). Due to extreme disturbance events (e.g. tornadoes) or potential field measurement errors, 1.8 % of plot observations were deemed outliers and removed from analyses (122 plots). Outlier removal protocol was based on 10 times the interquartile range such that a plot having either coarse woody debris carbon estimates > 43.7 tonnes ha^-1 or fine woody debris carbon estimates > 33.3 tonnes ha^-1 was excluded from study analyses. The field data used in this study may be accessed at [28].

The Köppen Climate Classification System, originally developed by Vldamir Köppen in 1900, is one of the most widely used for classifying the world's climatic regions [33-36]. Since the map's early introduction, numerous geographers have updated/modified it to the current version used in this study (United States map, see [37]). The modified Köppen classification uses three letters corresponding to average annual precipitation, average monthly precipitation, and average monthly temperature to divide the world into six major climatic regions of which only four are applicable to the conterminous United States. The first letter of the classification scheme refers to very broad climatic zones (e.g. equatorial or polar), the second letter denotes the seasonality of precipitation (e.g. summer dry or fully humid), and the third letter denotes the air temperature (e.g., hot or cool summer) (Table 3).

**Table 3:** Modified Köppen's climatic classification regions and subgroups used in this study

Climatic region	Climatic region description	Associated United States forest ecosystem/region
BS	Arid steppe	Intermountain dry grasslands
BW	Arid desert	Southwestern deserts
Cs	Warm temperate with dry summer	Chaparral
Cf	Warm temperate, fully humid	Southeastern mixed forests
Ds	Snow with dry summer	Northern Rockies
Dw	Snow with dry winter	Northern Rockies
Dfa	Snow, fully humid, hot summer	Mid-Atlantic
Dfb	Snow, fully humid, warm summer	Upper midwest and northeast
Dfc	Snow, fully humid, cool summer	Extreme northern boreal
H	Highland	High elevation alpine

Six climatic variables were correlated with coarse and fine woody debris carbon stocks in this study: PRECIP, TMAX, TMIN, MOIST, VAR, and EVAP. Data for the PRECIP, TMAX, and TMIN were obtained from the Parameter-elevation Regressions on Independent Slopes Model (PRISM) dataset (4-km grid cell size; PRISM Group [38]). Each of these three variables is represented by a 30-year climate normal. As such, annual precipitation is the mean annual total precipitation from 1971 to 2000. TMAX and TMIN are the mean daily temperature extremes for that period. Emerging research has indicated that certain forest attributes, such as standing live tree biomass, may be more correlated with certain monthly minimums/maximums (e.g., minimum January temperature) [39]. However, as little research has been conducted to determine which climatic summaries are most highly correlated with forest dead wood attributes, our study utilized annual summaries as starting point.

The MOIST, VAR, and EVAP climatic variables are derived from a revised Thornthwaite global climate classification (4-km grid cell size, [35]). MOIST, an indication of water surplus or deficit, is intended to indicate vegetation water availability. EVAP is a surrogate for the thermal energy available in a given location. VAR is derived from the MOIST variable and indicates whether seasonal variability is caused by temperature, precipitation, or a combination of both. The three variables for the revised Thornthwaite classification were derived from global temperature and precipitation datasets available from the University of Delaware Center for Climatic Research [40,41].

### Analysis

Coarse and fine woody debris carbon contents were estimated using a combination of line-intersect volume per unit area estimators and conversion factors for biomass and carbon [3]. Line-intersect sampling estimators were used to determine volume per unit area estimates for sample plots based on sub-plot transects. These volume per unit area estimators are simply equations to determine the volume that a given sample of coarse woody debris pieces represent for an entire area of interest. The shorter the sample transect or coarse woody debris piece the larger the volume that piece represents on a per unit area basis. Estimates of volume were first converted to biomass and then to carbon using conversion constants [3,31,42]. Carbon storage in coarse woody debris (C_CWD_) (tonnes ha^-1) was calculated using Eq. (1):

(1) $$C_{CWD}=\sum_{i=1}^{n}(c_{i}G_{i})\left[\left(\frac{\pi }{2L}\right)\left(\frac{V_{i}}{l_{i}}\right)f\right]$$

where *n *is the number of pieces, *c*_*i *_is the proportion of *C *in the mass of the *ith *piece, *f *is the conversion factor for unit-area values (10,000), *G*_*i *_is the estimated bulk density (g m^-3^) of the *ith *piece reduced by a modeled decay reduction factor, *L *is the total length of the transect corrected for slope (m), *V*_*i *_is the volume of the *ith *coarse woody piece (m^3^), and *l*_*i *_is the length of the *ith *piece in meters [3]. Birdsey [42] provides mean conversion factors (c) for both softwood (0.521) and hardwood species (0.491). Waddell [31] provides decay reduction factors for various coarse woody debris decay stages for reducing the specific gravity of coarse woody debris pieces based on the state of decay.

Carbon storage in fine woody debris (C_FWD_) (tonnes ha^-1) was calculated using Eq. (2):

(2) $$C_{FWD}=\sum_{i=1}^{n}\frac{\left(G_{i}a_{i}c_{i}sk\right)}{L}t_{i}{\bar{d}}_{i}^{2}$$

where *n *is fine woody debris size class (medium or large), *G*_*i *_is the bulk density (g m^-3^) of the *ith *class, *a*_*i *_is the nonhorizontal lean angle correction factor for *ith *class, *c*_*i *_is the proportion of carbon in the *ith *class, *s *is the slope correction factor because fine woody debris is measured along a slope-distance transect, *k *is a constant representing both unit conversion and a constant for fine woody debris piece lengths (1.234), *L *is the slope length of the transect (m), *t*_*i *_is the number of pieces of fine woody debris in the *ith *size class, and *d*_*i *_is the mean diameter (cm) of pieces within size class *i*. Because species data are not collected for fine woody debris, values of *G*, *c*, and *a *were based on the forest type assessed during phase 2 measurements [3]. Carbon storage for the smallest fine woody debris size class (< 0.64 cm) was not included in plot totals because this stock is included in forest floor measurements of litter.

The mean and standard errors for coarse and fine woody debris carbon stocks were estimated by Köppen climatic region/subregion. Köppen climatic subregions were collapsed into regions if sample sizes were insufficient. The significance of differences in mean coarse and fine woody debris carbon stocks between Köppen's classes was tested using an analysis of variance (ANOVA). Differences in means among climatic regions were deemed significant at the 0.05 level. To assess the relationship between coarse and fine woody debris carbon stocks and climatic variables and to elucidate dead wood trends between Köppen's classes, Pearson's correlations coefficients and associated p-values were determined and displayed in a correlation matrix. Additionally, a linear regression was conducted between the dependent variable of total woody detritus stocks and the independent climatic variables of TMAX and PRECIP.

## List of abbreviations

PRECIP: average annual precipitation; TMAX: mean annual maximum temperature; TMIN: mean annual minimum temperature; MOIST: moisture index; VAR: variability cause; EVAP: potential evapotranspiration; FIA: Forest Inventory and Analysis

## Competing interests

The authors declare that they have no competing interests.

## Authors' contributions

CWW designed the study, conducted the majority of analyses, and wrote the manuscript, GCL acquired all meteorological data layers and assisted with Geographic Information System analytical operations. Additionally, GCL provided interpretations of study results. All authors read and approved the final manuscript.
